# Clinical features and imaging markers of small vessel disease in symptomatic acute subcortical cerebral microinfarcts

**DOI:** 10.1186/s12883-022-02824-w

**Published:** 2022-08-23

**Authors:** Wendan Tao, Yajun Cheng, Wen Guo, William Robert Kwapong, Chen Ye, Bo Wu, Shuting Zhang, Ming Liu

**Affiliations:** 1 Center of Cerebrovascular Disease, Department of Neurology, West China Hospital, Sichuan University, Sichuan Province, No. 37 Guo Xue Xiang, Chengdu, 610041 People’s Republic of China; 2 Centre for Clinical Brain Sciences, University of Edinburgh, Edinburgh, UK

**Keywords:** Microinfarcts; recent small subcortical infarcts; small vessel disease, Acute lacunar stroke, DWI, Outcomes

## Abstract

**Background:**

As currently defined, recent small subcortical infarcts (RSSI) do not have a lower size boundary, and the smallest diffusion-weighted imaging (DWI) infarcts, which we term acute subcortical cerebral microinfarcts (As-CMI) with lesion diameter less than 5 mm, might have clinical implications distinct from RSSI. We aimed to investigate the distinct characteristics of As-CMI as compared to the larger size of RSSI regarding vascular risk factors, clinical manifestation, radiological markers of SVD distribution, and outcomes.

**Methods:**

In a consecutive cohort, patients were selected with a magnetic resonance DWI-confirmed RSSI between January 2010 and November 2020. We measured axial infarct diameter and classified patients into two groups: The As-CMI group (diameter < 5 mm) versus the Larger RSSI group (diameter 5-20 mm). Clinical variables, including vascular risk factors, clinical symptoms/signs, lesion locations, and radiological markers of cerebral small vessel disease (SVD) on MRI were analyzed between the two groups. Patients were followed up for 12 months and functional outcomes were measured by the modified ranking scale (mRS).

**Results:**

In a total of 584 patients with RSSI, 23 (3.9%) were defined as As-CMI. The most common neurological deficits with As-CMI were hemiparalysis (*n* = 20), followed by central facial/lingual palsy (*n* = 10) and hemidysesthesia (*n* = 10). Most As-CMIs were located in the basal ganglia (*n* = 11), followed by the thalamus (*n* = 5) and centrum semiovale (*n* = 4). No different regional distributions and symptoms/signs frequencies were found between the two groups except for a lower percentage of dysarthria in the As-CMI group (*p* = 0.008). In a multivariate analysis, patients with As-CMI were independently associated with the presence of lacunes (adjusted odds ratio [aOR] 2.88; 95% confidence interval [CI] 1.21–6.84), multiple lacunes (aOR 3.5, CI 1.29–9.48) and higher total SVD burden (aOR 1.68, CI 1.11–2.53). Patients with As-CMI did not show a better functional outcome after 12 months of follow-up.

**Conclusions:**

Patients with As-CMI had a non-specific clinical profile but a higher burden of SVD, indicating As-CMI might be s sign of more severe small vascular injury. Whether its vascular features are associated with worse cognitive outcomes requires further investigation.

## Introduction

Cerebral microinfarcts (CMIs) are small lesions presumed to be of ischemic origin [1]. The lesions, small and often not visible to the naked eyes on autopsy, have been found incidentally in older individuals with cognitive impairment or other manifestations of cerebrovascular disease, indicating damage to brain structure and a poor clinical outcome [2, 3].

CMIs in the acute stage can be detected as small incidental diffusion-weighted imaging (DWI) lesions. DWI has been verified with high sensitivity in detecting very small infarcts, including those 1–2 mm in diameter in any brain parenchymal location [4]. Due to the blooming effects, the maximum diameter of acute DWI might overestimate the true infarct size [5]. The defined diameter of acute CMI varied among studies, from a few mm to ≤ 10 mm [6]. The majority of studies on incidental small DWI lesions did not report their actual size and usually adopt a cut-off with a maximum axial diameter of less than 5 mm [7, 8]. Based on available evidence, experts propose a size criterion of less than 5 mm for an acute cerebral microinfarct (A-CMI) [1]. However, confusion arises when diagnosing a small DWI lesion in the sub-cortex of ischemic stroke patients. If perforating arterioles are affected, it will cause a recent small subcortical infarct (RSSI), formerly termed ‘acute lacunar stroke’. The STandards for ReportIng Vascular changes on nEuroimaging (STRIVE) criteria for RSSI applied an upper size cutoff of 20 mm, a criterion designed to identify lacunar infarcts in their acute stage, without providing a lower size cutoff [9]. As currently defined, RSSI does not have a lower size boundary, and the smallest DWI infarcts, which we term acute subcortical cerebral microinfarcts (As-CMI), might have clinical implications distinct from RSSI imaging characteristics of small DWI hyperintense lesions in vivo have been reported in patients with cerebral amyloid angiopathy, stroke, or memory loss [10–14]. These studies reported that the small DWI hyperintense lesions were associated with magnetic resonance imaging markers of cerebral small vessel disease (SVD), such as white matter hyperintensities, cerebral microbleeds, and enlarged perivascular spaces, but the association between different sizes of small subcortical DWI lesions and SVD neuroimaging markers has not been thoroughly investigated. Besides in the sub-cortex, the acute microinfarct could act as lacunar stroke syndrome, but whether it has similar symptoms/signs distribution or better outcome compared to larger RSSI is still unknown. A better understanding of the varied clinical features of As-CMI and larger RSSI could extend emerging literature from previous studies and provide further evidence concerning the diagnostic classification of acute SVD.

Hence, we aimed to clarify the distinct characteristics of As-CMI as compared to the larger size of RSSI regarding vascular risk factors, clinical manifestation, radiological markers of SVD distribution, and outcomes.

## Methods

### Patients

**Fig. 1:**
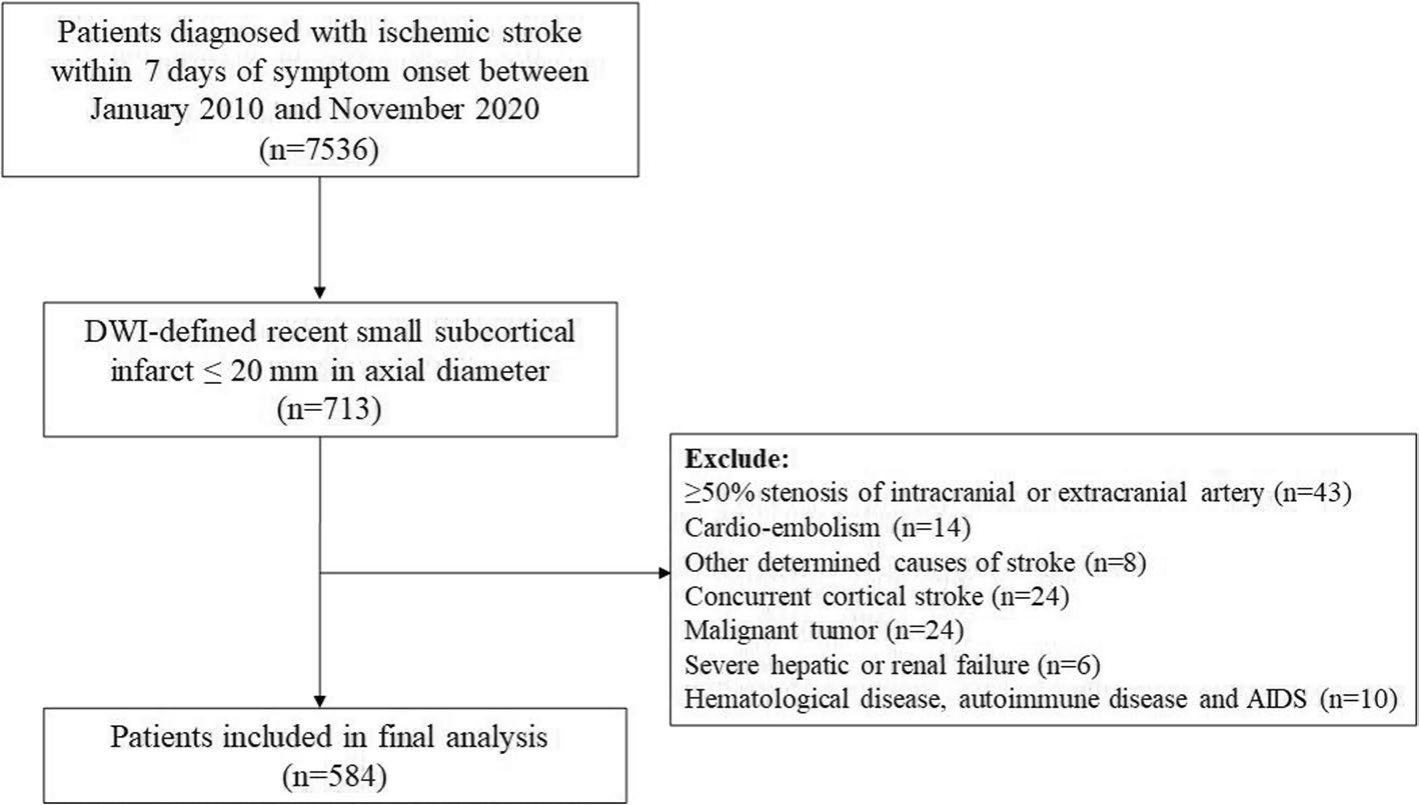
The flow diagram of patients’ selection

A total of 7536 patients with acute ischemic stroke were consecutively recruited from the Chengdu stroke registry between January 2010 and November 2020. All patients with acute ischemic stroke or transit ischemic stroke onset within 7 days were admitted to the neurology department. Patients with symptomatic magnetic resonance DWI-confirmed recent small subcortical infarcts of ≤ 20 mm in axial diameter were identified [9]. Exclusion criteria were RSSI lesion with an embolic source (cardioembolism or large-artery atherosclerosis) or other undetermined cause of stroke; concurrent cortical stroke; malignant tumor, severe hepatic or renal failure; hematological disease or autoimmune disease, and AIDS. Cardioembolism was defined as a cardiac source of high-risk and medium risk for a possible or probable diagnosis of cardioembolic stroke (e.g. AF). Large-artery atherosclerosis was defined as patients who have clinical and brain imaging findings of either significant stenosis (> 50%) or occlusion of a major brain artery or branch cortical artery, presumably due to atherosclerosis [15, 16]. Finally, 584 patients with symptomatic magnetic resonance DWI-confirmed recent small subcortical infarcts of ≤ 20 mm in axial diameter were included. The patients’ selection process is shown in Fig. 1.

The study was approved by the Medical Ethics Committee of West China Hospital, Sichuan University. The ethical principles involved in this research were strictly by the “Declaration of Helsinki”. The requirement for written informed consent was waived by the Medical Ethics Committee of West China Hospital, Sichuan University because of the retrospective study design.

### Data collection

In-hospital data was obtained through medical records and interviews with patients or their families [17]. Demographic characteristics include age, sex, time from symptom onset to MRI, prior modified Rankin scale (mRS) [18], initial stroke severity (assessed by the national institutes of health stroke scale, NIHSS) [19], diastolic and systolic blood pressure on admission, and vascular risk factors include hypertension, diabetes mellitus, hyperlipidemia, valvular heart disease, coronary artery disease, previous stroke, transient ischemic attack (TIA), alcohol abuse and smoking.

Clinical manifestations were systematically evaluated and defined based on the American Stroke Association’s published stroke warning signs and the most common lacunar syndromes [20, 21], with some modifications. A trained medical student blinded to the imaging results performed a chart review and completed a standardized form to obtain information regarding the symptoms/signs of acute stroke and the symptoms/signs that prompted the patients to seek medical attention. A neurologist was consulted if uncertain data were encountered. Nine categories were classified: disturbed consciousness (eg. confusion/delirium; somnolence; stupor; coma), speech disturbance (eg. aphasia; dysarthria), central facial/tongue paralysis, eye movement disorders (eg. oculomotor nerve palsy; gaze palsy), hemiparalysis, hemidysesthesia, dysphagia, ataxia, and complaint of dizziness/vertigo.

Functional outcome was measured by a modified Rankin scale (mRS) score [22], a 7-level ordered categorical scale (0 = independent, 6 = dead), through telephone interviews at 3 and 12 months after ischemic stroke.

### Neuroimaging

MRI was performed according to a standardized protocol as part of routine clinical assessments. Imaging was performed on a 3-T MR scanner with an acquisition that consisted of T1 and T2-weighted, fluid-attenuated inversion recovery (FLAIR), axial trace DWI with b-values of 0 and 1,000, and apparent diffusion coefficient (ADC) sequences. The MRI parameters are: T1 (repetition time [TR] 1530 ms; echo time [TE] 9.2 ms); T2 (TR 4,000 ms; TE 93 ms); FLAIR (TR 5000 ms; TE 93 ms); diffusion tensor imaging (TR 4880 ms; TE 77 ms).

#### Assessment of As-CMI and Larger RSSI in RSSI patients

**Fig. 2:**
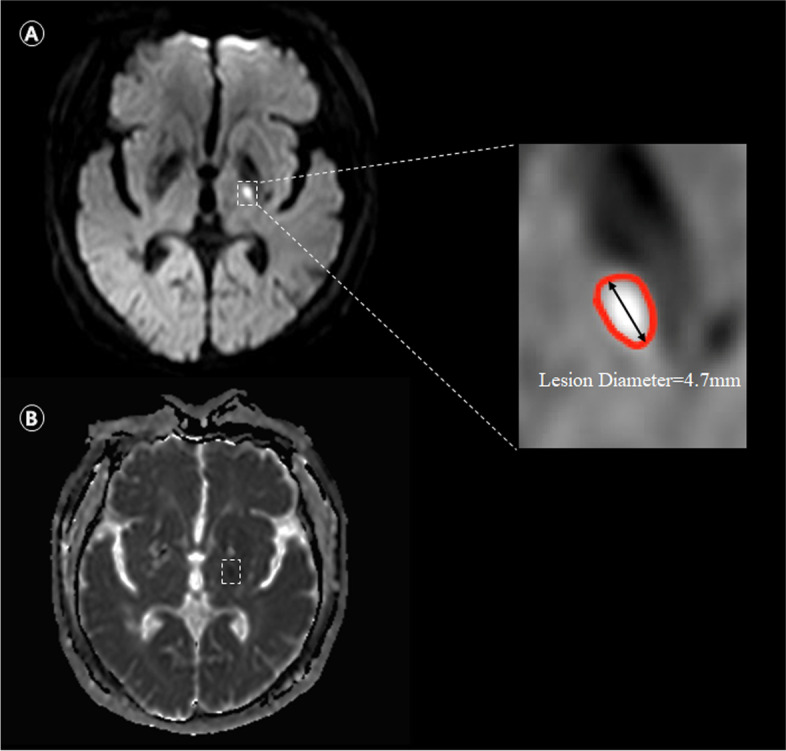
Illustrative image of the acute subcortical cerebral microinfarcts (As-CMI). Subcortical As-CMI detected on 3 T MRI in a 55-year-old man with sudden speech disturbance onset lasting more than 24 h. Neurological deficits assessed by NIHSS score was 3. Diffusion-weighted imaging showed a hyperintense subcortical lesion with longest diameter in axial orientation of 4.7 mm ( Dashed box in **A**), with a corresponding hypointense on ADC (Dashed box in **B**)

Two experienced raters blinded to demographic and clinical data independently reviewed the MRI studies to identify RSSI on MRI. RSSI was defined as hyperintense lesions on DWI in regions supplied by a penetrating artery, with a corresponding reduced diffusivity on ADC maps in the subcortical area, with a diameter ≤ 20 mm on axial sections. We chose the slice which showed the largest lesion size and measured the longest diameter in axial orientation. Of note, according to the proposed detection criteria for As-CMI [1], few very small DWI lesions had a corresponding isointense signal in ADC at the same location. Subcortical lesion location was classified into four regions (basal ganglia, centrum semiovale, thalamus, and pons). We measured axial infarct diameter and classified two groups: As-CMI (less than 5 mm, shown in Fig. 2) and Larger RSSI (greater than 5 mm and less than 20 mm).

#### Assessment of SVD MRI markers

Lacunes, white matter hyperintensity (WMH), and enlarged perivascular spaces EPVS in basal ganglia (BG-EPVS) and centrum semiovale (CSO-EPVS) were rated according to the STRIVE consensus criteria [9]. Lacunes were defined as rounded or ovoid fluid-filled cavities in subcortical regions, hyperintensities (diameter range 3-15 mm) on T2-weighted sequence with the corresponding hypointensity or with a hyperintense rim on FLAIR. WMH was characterized on FLAIR images using the Fazekas scale. The severity of WMH was rated (0 to 3) separately in deep and periventricular regions, with the sum of the scores providing a total WMH score. Extensive WMH was defined as deep white matter hyperintensity (DWMH) (score ≥ 2) or periventricular white matter hyperintensity (PWMH) (score 3). EPVS were measured as linear fluid-filled space hyperintensities on a T2-weighted sequence with a diameter less than 3 mm followed the typical course of a vessel in the grey or white matter and counted separately in the basal ganglia (BG) and centrum semiovale (CS), according to a 3-category ordinal scale (0–10, 10–25, and > 25). To qualify the cumulative effect of small vessel injury, the total burden of SVD was assessed on an ordinal scale (range 0 to 3), where 1 point was awarded to each of the following: the presence of any lacune, PWMH score of 3 and/or DWMH score ≥ 2, and the number of BG EPVS > 10.

MRI images were visually inspected with software (RadiAnt DICOM Viewer1.0.4.4439; Medixant Ltd, Poznan, Poland). SVD markers and As-CMI were evaluated separately by raters (C.Y.J and G.W) blind to clinical data. A second rater (W.D.T) evaluated a random sample of 50 patients to assess inter-rater agreement for presence of lacunes (kappa 0.82, *P* < 0.001), EPVS in CSO (kappa 0.622, *P* < 0.001), EPVS in BG (kappa 0.71, *P* < 0.001), severity of WMH (kappa 0.73, *P* < 0.001) and As-CMI (kappa 0.87, *P* < 0.001).

### Data analysis

Continuous variables were expressed as mean ± standard deviation (SD) and categorical variables as frequencies and percentages. Comparison between the groups was performed using a chi-squared test or Fisher exact test for categorical variables, and an independent sample *t*-test or Mann–Whitney *U* test for continuous variables, as appropriate. Binary logistic regression methods were used to analyze the odds (with a 95% confidence interval) of As-CMI and SVD imaging markers. All *p* values were two-sided and values < 0.05 were considered statistically significant. All analyzes were performed using SPSS (version 23, SPSS Inc).

## Results

Of the 584 patients finally included, 23 (3.9%) were defined as As-CMI. Five patients in the Larger RSSI group had multiple DWI small lesions.

### Demographic and vascular risk factors in patients of As-CMIs and Larger RSSI

The mean age of patients in the study population was 62.2 (SD12.4) years and 71.9% were male. A higher frequency of a history of coronary artery disease was observed in the As-CMI subjects (*p* = 0.020). However, no significant differences were seen in other demographic or vascular risk factors between As-CMI and Larger RSSI groups (Table 1).

**Table 1:** Demographics and vascular risk factors between As-CMI and Larger RSSI groups

Characteristics	As-CMI, *n* = 23	Larger RSSI, *n* = 561	*P* value
**Age,yr**	67.2 ± 13.0	62.0 ± 12.4	0.050
**Male**	15(65.2)	405(72.2)	0.466
**Interval between stroke onset to imaging scanning**			0.590
< 24 h	1(4.3)	9(1.6)	
1-7 days	19(82.6)	467 (83.2)	
8-14 days	3(13.0)	85(15.2)	
**Prestroke mRS 0–1, n (%)**	20(87.0)	542(96.6)	0.068
**NIHSS at admission**	2(1–4)	3(2–5)	0.080
**SBP (mmHg)**	161.2 ± 21.7	160.1 ± 22.6	0.826
**DBP (mmHg)**	91.5 ± 14.5	93.9 ± 15.4	0.462
**Vascular risk factors**			
**Hypertension**	17(73.9)	407(72.5)	0.886
**Diabetes Mellitus**	8(34.8)	206(36.7)	0.850
**Hyperlipidemia**	5(21.7)	206(36.7)	0.143
**Coronary artery disease**	4(17.4)	24(4.3)	0.020
**Previous ischemic stroke**	5(21.7)	51(9.1)	0.059
**Current drinking**	4(17.4)	158(28.2)	0.258
**Current smoking**	8(34.8)	225(40.2)	0.605

Data are *n* (%), mean (SD) or median (IQR)

Abbreviation: *mRS*, Modified ranking scale, *NIHSS*, National institutes of health stroke scale; *SBP*, Systolic blood pressure, *DBP*, Diastolic blood pressure

#### Clinical manifestations and lesion location in patients of As-CMI and Larger RSSI

**Fig. 3:**
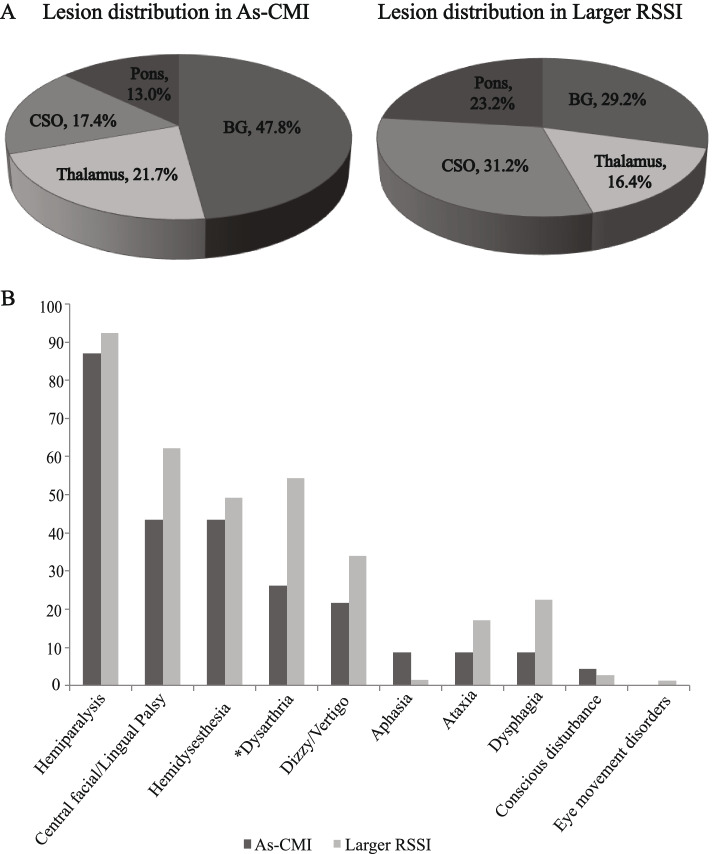
The distribution of lesion location and frequency of single clinical symptom/sign between As-CMI and Larger RSSI groups. Basal ganglia (BG), thalamus, central semiovale (CS) and brainstem constituted 47.8%, 21.7%, 17.4% and 13% respectively in As-CMI subjects. No significant difference of lesion distribution was found between the two groups (**A**). In As-CMI, the most common neurological deficits was hemiparalysis (87%), followed by central facial/lingual palsy (43.5%) and hemidysesthesia (43.5%). No significant difference of symptoms/signs was found between the two groups except for a lower percentage of dysarthria in the As-CMI subjects (**B**). *Dysarthria (As-CMI 26.1% vs. Larger RSSI 54.4%, *p* = 0.008)

In the As-CMI group, hemiparalysis (*n* = 20) was the most common neurological deficit, followed by central facial/lingual palsy (*n* = 10) and hemidysesthesia (*n* = 10). Most As-CMI lesions were in the basal ganglia (*n* = 11), followed by the thalamus (*n* = 5) and centrum semiovale (*n* = 4). Patients with As-CMI and Larger RSSI did not differ in clinical manifestations and lesion location generally, except for a lower percentage of dysarthria in the As-CMI (*p* = 0.008) (Fig. 3).

#### SVD neuroimaging markers in patients of As-CMI and Larger RSSI

After adjusting for age, sex, and coronary artery disease, As-CMI was associated with the presence of lacunes (odds ratio [OR] 2.88; 95% confidence interval [CI] 1.21–6.84), multiple lacunes (OR 3.5, CI 1.29–9.48) and higher total SVD burden (OR 1.68, CI 1.11–2.53). No association was found between As-CMI and the severity of WMH and BG-EPVS or CSO-EPVS (Table 2).

**Table 2:** Neuroimaging markers of SVD between As-CMI and Larger RSSI groups

	**As-CMI,** ***n* = 23**	**Larger RSSI,** ***n* = 561**	Fully-adjusted* OR(95%CI)	***P* value**
**Presence of Lacunes**	14(60.9)	188(33.5)	2.88(1.21–6.84)	0.016
**Lacunes number**				0.039
0	9(39.1)	373(66.5)	Reference	
1	6(26.1)	102(18.2)	2.34(0.81–6.79)	0.115
> 1	8(34.8)	86(15.3)	3.50(1.29–9.48)	0.013
**The severity of WMH**				0.060
0–2	11(47.8)	310(55.3)	Reference	
3–4	3(13.0)	154(27.5)	0.52(0.14–1.90)	0.323
5–6	9(39.1)	97(17.3)	2.29(0.90–5.80)	0.081
**The severity of EPVS in BG**				0.391
≤ 10	5(21.7)	227(40.5)	Reference	
11–20	13(56.5)	220(39.2)	2.07(0.68–6.28)	0.198
> 20	5(21.7))	114(20.3)	1.38(0.34–5.59)	0.645
**The severity of EPVS in CSO**				0.559
≤ 10	4(17.4)	76(13.5)	Reference	
11–20	10(43.5)	287(51.2)	0.51(0.14–1.75)	0.285
> 20	9(39.1)	198(35.3)	0.56(0.15–2.05)	0.383
**Total CVSD Burden**	2(1–3)	1 (0–2)	1.68(1.11–2.53)	0.013

*Abbreviation:* *SVD*, Cerebral small vessel disease; *WMH*, White matter hyperintensity; *EPVS*, Enlarged perivascular spaces; *BG*, Basal ganglia, *CSO*, Centrum semiovale

^*^Adjusted for age, sex, coronary artery disease

#### Follow-up

Data for 3-month and 1-year follow-ups were obtained from 526 of 584 (90.0%) and 513 of 584 (87.8%) eligible patients. The As-CMI group did not show a better functional outcome at 3-months follow-up (median mRS 1.0 vs.1.0; *p* = 0.924) and 12-months (median mRS 0 vs.1.0; *p* = 0.791) compared to the Larger RSSI group. Twenty patients (86.9%) in the As-CMI group completed 3 and 12-month follow-ups, of whom 8 patients (40%) had an improvement, 11 unaltered, and 1 unfavorable shift in mRS score, which were comparable to the Larger RSSI group (*p* = 0.820, Table 3).

**Table 3:** Outcomes at 3 and 12 months for As-CMI and Larger RSSI groups

	**As-CMI**	**Larger RSSI**	***P* value**
**3-months follow-up, n(%)**	506(90.1)	20(86.9)	
mRS score, median(IQR)	1(0–2)	1(1–2)	0.924
**12-months follow-up, n(%)**	492(87.7)	21(91.3)	
mRS score, median(IQR)	0(0–1.5)	1(0–1)	0.791
**mRS shifts more than 1 point after12-months follow-up, n(%)**	484(86.2)	20(86.9)	0.820
Favorable, n(%)	161(33.3)	8(40.0)	
Unaltered, n(%)	294(60.7)	11(55.0)	
Unfavorable, n(%)	29(6.0)	1(5.0)	

## Discussion

To the best of our knowledge, the present study is the first to examine As-CMI in a large series of consecutive patients in whom DWI has also shown RSSI. In our current study, we found that As-CMI was identified in 3.9% of RSSI patients. We found a similar distribution among risk factors, common symptoms/signs as well as lesion location. Secondly, we assessed the association between As-CMI and SVD markers and observed that the As-CMI was associated with lacunes and a higher total SVD burden. Thirdly, we demonstrated that patients with As-CMI did not have a better functional outcome compared to patients with Larger RSSI.

The mean age in our study was 62.2 years which is congruent with previous reports on the Asian race [23, 24]. Nonetheless, the mean age of previous reports on subcortical lacunes in the Caucasian race was higher [25, 26]. This may be due to the different population study design (i.e. in terms of age range), classification of lacunes and/or lacunar infarction, and race. In addition, a higher frequency of a history of coronary artery disease (CAD) was observed in subjects with As-CMI compared to the larger RSSI group. The epidemiology of SVD and its consequences on the heart is not well understood [27]. It could be explained by a higher mean age in the As-CMI group that could cause an age-related high incidence of CAD. Furthermore, the vascular anatomy of the heart and the brain is quite similar, with arteries on the surface and penetrating arteries that provide tissue perfusion [28]. Thus, further studies are required to investigate the association between small vessel disease in the heart and brain and CAD.

In our series, dichotomization of RSSI according to the axial diameter (< / ≥ 5 mm) did not result in quite different regional distributions and symptoms/signs frequencies. In patients with symptomatic As-CMI, nearly half of the lesions were located in the basal ganglia, followed by the thalamus, centrum semiovale (CSO), and brainstem. In addition, hemiparalysis was the most common neurological deficit, followed by central facial/lingual palsy and hemidysesthesia. These are common symptoms in acute lacunar stroke according to the widely used OCSP classification [19]. In other words, the clinical symptoms/signs poorly discriminate the size of subcortical infarcts. Our findings showed that even the small lesion size of As-CMI could cause overt neurological symptoms. Noticeably, As-CMI could also contribute to other low-frequency symptoms such as dysphagia, aphasia, or ataxia.

Another novel finding was the association between subcortical As-CMI and the presence of multiple lacunes and a high total SVD burden. Previous studies reported a higher SVD burden in patients with RSSI or As-CMI who presented with possible vascular cognitive impairment [7, 29]. However, the relation between different RSSI sizes and SVD burden was not explored. As-CMI located in the white matter mostly occurs in relationship to distal branches of perforating arteries [30]. The increased frequency of lacunes and SVD burden in As-CMI showed that As-CMI might be a significant sign of active small vessel disease. The anatomical and hemodynamic characteristics of these smaller and distal branches of medullary or lenticulostriate arteries may partly explain an increased vulnerability of these territories to hypoperfusion injury and other pathophysiological mechanisms such as arteriosclerosis, endothelial injury, or blood–brain barrier dysfunction [30, 31]. Interestingly, in a novel whole-brain vessel-wall MRI study, patients with symptomatic single subcortical infarction but without relevant MCA disease were included. Although lesion size was not measured in the study, it is found that superiorly distributed MCA plaques at the lenticulostriate arteries origin (LSA) are associated with morphological changes in the LSA [32]. Whether the smaller size of the lesion would reflect worse morphological changes in the supplying artery and cause heavier SVD damage needs to be verified.

In long-term follow-up, our data did not show that As-CMI patients had a better functional outcome and recovery. The mRS score we applied is good at measuring the degree of disability or dependence in the daily activities of patients after stroke. However, the majority of lacunar infarcts would cause minor stroke-related deficits. Among lacunar strokes, the mRS might not be a subtle tool to evaluate the degree of rehabilitation. A previous study indicated brain frailty was associated with a worse cognitive score with a stronger effect in lacunar stroke [33]. As-CMI was found to increase odds of lacunes and SVD burden, implying a correlation with a worse cognitive outcome that merits further investigation.

Our study has several strengths such as the large hospital-based sample with MR-DWI, comprehensive evaluation of clinical symptoms/signs, and the assessment of SVD markers and outcomes. However, we would like to acknowledge some limitations. First, there is an ongoing discussion on whether different RSSI lesion sizes are associated with a distinct etiology. Thus, the present study excluded patients with potential embolic sources or undetermined causative mechanisms to focus on small vessel arteriopathy. Second, cerebral microbleeds were not included in the total SVD burden evaluation since a large proportion of patients did not undergo gradient-echo T2*-weighted imaging. Third, because of its retrospective nature, we cannot avoid selection bias. Also, the number of As-CMI is relatively small and until now the evaluation of imaging markers of As-CMI and SVD are mainly based on neurologists.

In conclusion, we add to the increasing body of evidence that patients with As-CMI had a non-specific clinical profile but a higher burden of SVD, indicating As-CMI might be s sign of more severe small vascular injury. Whether its vascular features are associated with worse cognitive outcomes requires further investigation.

## Data Availability

Original data to support the results of this study are not publicly available due to privacy reasons of patients, but are available from the corresponding author upon reasonable request.

## Abbreviations

As-CMI: Acute subcortical microinfarct
RSSI: Recent small subcortical infarcts
DWI: Diffusion-weighted imaging
STRIVE: The STandards for ReportIng Vascular changes on nEuroimaging
MRI: Magnetic resonance imaging
FLAIR: Fluid-attenuated inversion recovery
ADC: Apparent diffusion coefficient
WMH: White matter hyperintensity
EPVS: Enlarged perivascular spaces
BG: Basal ganglia
CSO: Centrum semiovale
mRS: Modified Ranking scale

## References

[1] van Veluw SJ, Shih AY, Smith EE, Chen C, Schneider JA, Wardlaw JM, Greenberg SM, Biessels GJ (2017). Detection, risk factors, and functional consequences of cerebral microinfarcts. Lancet Neurol.

[2] Smith EE, Schneider JA, Wardlaw JM, Greenberg SM (2012). Cerebral microinfarcts: the invisible lesions. Lancet Neurol.

[3] Brundel M, de Bresser J, van Dillen JJ, Kappelle LJ, Biessels GJ (2012). Cerebral microinfarcts: a systematic review of neuropathological studies. J Cereb Blood Flow Metab.

[4] Keir SL, Wardlaw JM (2000). Systematic review of diffusion and perfusion imaging in acute ischemic stroke. Stroke.

[5] Del Bene A, Makin SD, Doubal FN, Inzitari D, Wardlaw JM (2013). Variation in risk factors for recent small subcortical infarcts with infarct size, shape, and location. Stroke.

[6] Ishikawa H, Ii Y, Shindo A, Tabei KI, Umino M, Ito AO, Matsuura K, Taniguchi A, Matsuyama H, Niwa A (2020). Cortical Microinfarcts Detected by 3-Tesla Magnetic Resonance Imaging: Differentiation Between Cerebral Amyloid Angiopathy and Embolism. Stroke.

[7] Ferro DA, van den Brink H, Exalto LG, Boomsma JMF, Barkhof F, Prins ND, van der Flier WM, Biessels GJ (2019). group T-Vs: **Clinical relevance of acute cerebral microinfarcts in vascular cognitive impairment**. Neurology.

[8] Oliveira J, Ay H, Shoamanesh A, Park KY, Avery R, Sorgun M, Kim GM, Cougo PT, Greenberg SM, Gurol ME (2018). Incidence and Etiology of Microinfarcts in Patients with Ischemic Stroke. J Neuroimaging.

[9] Wardlaw JM, Smith EE, Biessels GJ, Cordonnier C, Fazekas F, Frayne R, Lindley RI, O'Brien JT, Barkhof F, Benavente OR (2013). Neuroimaging standards for research into small vessel disease and its contribution to ageing and neurodegeneration. Lancet Neurol.

[10] Prabhakaran S, Gupta R, Ouyang B, John S, Temes RE, Mohammad Y, Lee VH, Bleck TP (2010). Acute brain infarcts after spontaneous intracerebral hemorrhage: a diffusion-weighted imaging study. Stroke.

[11] Gregoire SM, Charidimou A, Gadapa N, Dolan E, Antoun N, Peeters A, Vandermeeren Y, Laloux P, Baron JC, Jager HR (2011). Acute ischaemic brain lesions in intracerebral haemorrhage: multicentre cross-sectional magnetic resonance imaging study. Brain.

[12] Menon RS, Burgess RE, Wing JJ, Gibbons MC, Shara NM, Fernandez S, Jayam-Trouth A, German L, Sobotka I, Edwards D (2012). Predictors of highly prevalent brain ischemia in intracerebral hemorrhage. Ann Neurol.

[13] Wang Z, van Veluw SJ, Wong A, Liu W, Shi L, Yang J, Xiong Y, Lau A, Biessels GJ, Mok VC (2016). Risk Factors and Cognitive Relevance of Cortical Cerebral Microinfarcts in Patients With Ischemic Stroke or Transient Ischemic Attack. Stroke.

[14] Wu B, Yao X, Lei C, Liu M, Selim MH (2015). Enlarged perivascular spaces and small diffusion-weighted lesions in intracerebral hemorrhage. Neurology.

[15] Adams HP, Bendixen BH, Kappelle LJ, Biller  J, Love BB, Gordon DL DL, Marsh EE (1993). Classification of subtype of acute ischemic stroke. Definitions for use in a multicenter clinical trial. TOAST. Trial of Org 10172 in Acute Stroke Treatment. Stroke.

[16] Ay H, Benner T, Arsava EM, Furie KL, Singhal AB, Jensen MB, Ayata C, Towfighi A, Smith EE, Chong JY (2007). A computerized algorithm for etiologic classification of ischemic stroke: the Causative Classification of Stroke System. Stroke.

[17] Liu J, Zheng L, Cheng Y, Zhang S, Wu B, Wang D, Zhang S, Tao W, Wu S, Liu M (2019). Trends in Outcomes of Patients With Ischemic Stroke Treated Between 2002 and 2016: Insights From a Chinese Cohort. Circ Cardiovasc Qual Outcomes.

[18] de Haan R, Limburg M, Bossuyt P, van der Meulen J, Aaronson N (1995). The clinical meaning of Rankin 'handicap' grades after stroke. Stroke.

[19] Brott T, Adams HP, Olinger CP, Marler JR, Barsan WG, Biller J, Spilker J, Holleran R, Eberle R, Hertzberg V (1989). Measurements of acute cerebral infarction: a clinical examination scale. Stroke.

[20] Nicol MB, Thrift AG (2005). Knowledge of risk factors and warning signs of stroke. Vasc Health Risk Manag.

[21] Micheli S, Corea F (2012). Lacunar versus non-lacunar syndromes. Front Neurol Neurosci.

[22] Bruno A, Shah N, Lin C, Close B, Hess DC, Davis K, Baute V, Switzer JA, Waller JL, Nichols FT (2010). Improving modified Rankin Scale assessment with a simplified questionnaire. Stroke.

[23] Hao Z, Chen Y, Wright N, Qin H, Turnbull I, Guo Y, Kartsonaki C, Sansome S, PeiPei, Yu C (2021). Natural history of silent lacunar infarction: 10-year follow-up of a community-based prospective study of 0.5 million Chinese adults. Lancet Reg Health West Pac.

[24] Nah HW, Kang DW, Kwon SU, Kim JS (2010). Diversity of single small subcortical infarctions according to infarct location and parent artery disease: analysis of indicators for small vessel disease and atherosclerosis. Stroke.

[25] Moretti R, Caruso P, Storti B, Saro R, Kassabian B, Sala A, Giannini A, Gazzin S (2020). Behavior in subcortical vascular dementia with sight pathologies: visual hallucinations as a consequence of precocious gait imbalance and institutionalization. Neurol Sci.

[26] Ling Y, Chabriat H (2020). Incident cerebral lacunes: A review. Journal of cerebral blood flow and metabolism : official journal of the International Society of Cerebral Blood Flow and Metabolism.

[27] Berry C, Sidik N, Pereira AC, Ford TJ, Touyz RM, Kaski JC, Hainsworth AH (2019). Small-Vessel Disease in the Heart and Brain: Current Knowledge, Unmet Therapeutic Need, and Future Directions. J Am Heart Assoc.

[28] Moretti R, Janjusevic M, Fluca AL, Saro R, Gagno G, Pierri A, et al. Common Shared Pathogenic Aspects of Small Vessels in Heart and Brain Disease. Biomedicines. 2022;10(5):1009. 10.3390/biomedicines10051009 PMC9138783 35625746

[29] Rudilosso S, Mena L, Esteller D, Olivera M, Mengual JJ, Montull C, Castrillo L, Urra X, Gomez-Choco M (2021). Higher Cerebral Small Vessel Disease Burden in Patients with White Matter Recent Small Subcortical Infarcts. J Stroke Cerebrovasc Dis.

[30] Moody DM, Bell MA, Challa VR (1990). Features of the cerebral vascular pattern that predict vulnerability to perfusion or oxygenation deficiency: an anatomic study. AJNR Am J Neuroradiol.

[31] Wardlaw JM, Smith C, Dichgans M (2019). Small vessel disease: mechanisms and clinical implications. Lancet Neurol.

[32] Jiang S, Yan Y, Yang T, Zhu Q, Wang C, Bai X, Hao Z, Zhang S, Yang Q, Fan Z (2020). Plaque Distribution Correlates With Morphology of Lenticulostriate Arteries in Single Subcortical Infarctions. Stroke.

[33] Appleton JP, Woodhouse LJ, Adami A, Becker JL, Berge E, Cala LA, Casado AM, Caso V, Christensen HK, Dineen RA (2020). Imaging markers of small vessel disease and brain frailty, and outcomes in acute stroke. Neurology.

